# Protective effect of *Moringa oleifera* leaf ethanolic extract against uranyl acetate-induced testicular dysfunction in rats

**DOI:** 10.1038/s41598-023-50854-2

**Published:** 2024-01-09

**Authors:** Sohair M. M. Ragab, Hailah M. Almohaimeed, Alshaimaa A. I. Alghriany, Nasser S. Abou Khalil, Elham A. Abd-Allah

**Affiliations:** 1 Laboratory of Physiology, Department of Zoology and Entomology, Faculty of Sciences, Assiut University, Assiut, Egypt; 2 Department of Basic Science, College of Medicine, Princess Nourah Bint Abdulrahman University, P.O. Box 84428, Riyadh, 11671 Saudi Arabia; 3 Department of Zoology and Entomology, Faculty of Science, Assiut University, Assiut, Egypt; 4 Department of Basic Medical Sciences, Faculty of Physical Therapy, Merit University, Sohag, Egypt; 5 Department of Medical Physiology, Faculty of Medicine, Assiut University, Assiut, 71526 Egypt; 6 Department of Zoology, Faculty of Science, New Valley University, El-Kharga, Egypt

**Keywords:** Environmental sciences, Endocrinology

## Abstract

Uranyl acetate (UA) is used in civilian and military applications, predisposing it to wide dispersion in ecosystems. Using high-performance liquid chromatography, gas chromatography–mass spectrometry, and 2,2-Diphenyl-1-picrylhydrazyl scavenging radical analysis, we confirmed that *Moringa oleifera* leaf ethanolic extract (MLEE) is rich in biologically active phytochemicals. Thus, this study aims to investigate the possible defensive effect of MLEE against UA-induced testicular dysfunction. To achieve this, rats were divided randomly and evenly into three groups for 14 days. The control group received no treatment, while the UA group received a single intraperitoneal injection of UA at a dose of 5 mg/kg BW dissolved in saline on the 12th day of the experiment, followed by no treatment the following day. The MLEE + UA group received daily oral administration of MLEE (300 mg/kg BW) dissolved in distilled water before exposure to UA intoxication. The disruption observed in the pituitary–gonadal axis of UA-intoxicated rats was characterized by a significant decrease in luteinizing hormone, follicle-stimulating hormone, testosterone, and estradiol 17beta levels. Additionally, there was a notable increase in malondialdehyde and a decrease in catalase, superoxide dismutase, reduced glutathione, and nitric oxide, accompanied by an up-regulation in the immuno-expression of nuclear factor-kappa B, indicating a disturbance in the redox balance. The TUNEL assay confirmed a substantial rise in apoptotic cell numbers in the UA group. Testicular histopathological changes, excessive collagen deposition, and reduced glycogen content were evident following UA exposure. However, supplementation with MLEE effectively countered these mentioned abnormalities. MLEE is proposed to combat the toxicological molecular targets in the UA-affected testis by restoring the balance between oxidants and antioxidants while obstructing the apoptotic cascade. MLEE contains an abundance of redox-stabilizing and cytoprotective phytochemicals that have the potential to counteract the mechanistic pathways associated with UA exposure. These findings encourage further research into other plausible protective aspects of *Moringa oleifera* against the UA challenge.

## Introduction

Male infertility poses a global concern, significantly impacting humanity by causing substantial psychological and social struggles, as well as incurring significant financial costs in healthcare sectors^1^. Testicular structures are particularly susceptible to free radical damage due to their high cellular replication, accelerated mitochondrial oxygen expenditure, and abundance of unsaturated lipids^2^. Therefore, incorporating antioxidant supplements may prove beneficial in counteracting the adverse effects of oxidative stress and promoting fertility.

Uranyl acetate (UA), a by-product of uranium enrichment processes, is commonly used in industrial and military applications and originates from nuclear power plant fuel^3^. Its combined chemical and radiological hazards, multiple exposure pathways, and accumulation in the food chain^4^ have drawn significant attention from scholars, particularly amid the increasing probability of nuclear disputes in conflict zones. Furthermore, the escalating demand for nuclear fuel raises concerns about increased UA leakage into the ecosystem^4^. While chelation therapies have potential in reducing UA accumulation in organs and expediting its removal^5^, they face various limitations such as poor tissue specificity, low efficiency, and the risk of inducing acid–base disturbances and acute nephritis^5–7^. These limitations have prompted researchers to explore natural-derived approaches for mitigating UA’s radiological hazards, given their broad safety and bioavailability^8^.

The *Moringa oleifera* (MO) tree is primarily cultivated for its diverse uses as an essential herb, valued for its nutraceutical and medicinal properties^9^. Various parts of MO, such as the roots, flowers, fruits, seeds, and leaves, have been traditionally employed to combat abdominal tumors, paralysis, helminthic bladder issues, prostate problems, sores, and skin infections^10^. MO leaves, abundant in antioxidants and cytoprotective natural agents, position it as a promising future strategy in mitigating abnormalities linked to cellular peroxidative damage and apoptosis^11,12^. The amelioration observed in testicular deformities, impaired spermatogenesis, disruptions in the sexual axis, redox imbalances, and increased apoptosis^13,14^ post-supplementation with MO extracts highlight their potential in addressing reproductive dysfunction.

A limited number of reports have suggested the role of *Moringa oleifera* leaf ethanolic extract (MLEE) as a radioprotective agent. MLEE shielded gamma-irradiated rats from redox imbalances, resulting in decreased relaxation time and increased electrical conductivity in bone marrow^15^. Furthermore, MLEE prevented lipid peroxidation and nuclear translocation of nuclear factor kappa B while restoring reduced glutathione levels in the livers of mice exposed to gamma radiation^16,17^.

However, as far as we are aware, there is currently no existing data on the protective effects of MLEE against UA-induced testicular toxicity. Therefore, this study aims to investigate this aspect by assessing changes in the plasma pituitary–gonadal hormonal profile, testicular redox balance, histological structure, TUNEL assay, and nuclear factor-kappa B (NF-κB) immuno-expression in male Wistar rats. This research seeks to elucidate the potential protective role of MLEE in countering UA-induced testicular damage, addressing a notable gap in current knowledge. In regions or communities affected by UA contamination in the environment, MLEE-based interventions might offer protective measures against potential reproductive health complications. It's important to note that while the findings on MLEE's protective effects against UA-induced testicular toxicity hold promise, further rigorous research, including clinical trials and safety assessments, is essential before its direct application in human health care. Regulatory approvals and comprehensive studies are necessary to validate its efficacy, safety, and appropriate dosages for human use.

## Materials and methods

### Chemicals

UA, with a purity ≥ 98% and a molecular weight 424.15 g/mol, was purchased from Sigma-Aldrich Company (St. Louis, MO, USA).

### Preparation of plant extract

Two kilograms of freshly harvested green MO leaves were purchased from the Faculty of Agriculture farm at Assuit University, Egypt. A voucher was retained at the Assiut University Herbarium in Egypt, and MLEE was prepared following the method outlined by Mousa et al.^18^.

### Gas chromatography–mass spectrometry analysis

The analysis was conducted at the Functional and Therapeutic Foods Laboratory, Faculty of Agriculture, Alexandria University, Egypt, utilizing a trace GC-TSQ mass spectrometer (Thermo Scientific, Austin, TX, USA). Plant-derived constituents were identified by matching their mass spectra to the mass spectral databases WILEY 09 and NIST^14^.

### High-performance liquid chromatography

The analysis of phenolic and flavonoid compounds was conducted using the HPLC apparatus (Agilent Series 1100) from Agilent, USA. This apparatus includes an auto-sampling injector, solvent degasser, two LC-pumps (Series 1100) with ChemStation software, and a UV/Vis detector set at 250 nm for phenolic acids and 360 nm for flavonoids. The analysis employed a C18 column (125 mm × 4.60 mm, 5 µm particle size). For phenolic acids, separation was achieved using a gradient mobile phase of two solvents: methanol as solvent A and acetic acid in water (1:25) as solvent B. The gradient program initiated with 100% B and maintained this concentration for the initial 3 min. Subsequently, the eluent A concentration was set at 50% for the following 5 min, increased to 80% for the subsequent 2 min, and then reverted to 50% for another 5 min at a detection wavelength of 250 nm. For flavonoids, separation utilized a mobile phase composed of acetonitrile (A) and 0.2% (v/v) aqueous formic acid (B) with an isocratic elution (70:30) program. The solvent flow rate was maintained at 1 ml/min, and separation occurred at 25 °C. Injection volumes were set at 25 μL.

### Evaluation of antioxidant activity by 1, 1- diphenyl-2-picryl hydrazyl radical scavenging method

Free radical scavenging activity of MLEE was measured by 1, 1- diphenyl-2-picryl hydrazyl (DPPH) according to González-Palma et al.^19^.

### Experimental protocol

A total of 18 male Wistar adult rats were utilized in this study. They were procured from the Egyptian Company for the Production of Vaccines, Sera, and Drugs, Egypt, and were bred under natural light/dark cycles at a temperature of 20–25 °C and a relative humidity of 55.0 ± 5.0%. The rats were provided with commercial pelleted feed and water ad libitum.

Following a one-week adaptation period, the rats were randomly divided into three groups for a 14-day study. The control group received no treatment. The UA group received a single intraperitoneal injection of UA at a dose of 5 mg/kg BW dissolved in saline^20^ on the 12th day of the experiment. The rats received no treatment on the subsequent day. The MLEE + UA group received daily oral administration of MLEE (300 mg/kg BW)^18^ dissolved in distilled water prior to UA intoxication.

### Collection of samples

At the end of the experimental period, blood samples were collected from the retro-orbital sinus after overnight fasting using EDTA-containing tubes. After centrifugation at 3000 rpm for 10 min, plasma was obtained and stored at − 20 °C for the measurement of biochemical parameters. The rats were euthanized by cervical dislocation under anesthesia induced by intraperitoneal injection of sodium thiopental. One testis was promptly excised and homogenized in 1 ml of 0.1 M phosphate buffer (pH 7.4) using the IKA Yellow line DI homogenizer (18 Disperser, Germany) to create a 10% w/v homogenate. The homogenates were then centrifuged at 10,000 rpm for 15 min, and the resulting supernatants were frozen at − 20 °C for the assessment of oxidant/antioxidant parameters. The other testis was fixed in 10% neutral buffered formalin for subsequent histopathological examination.

### Biochemical measurements

Luteinizing hormone (LH), follicle-stimulating hormone (FSH), testosterone, and estradiol 17beta (E2) were measured by Cobas E601 Immunology Analyzer, Roche Diagnostics, USA. The levels of malondialdehyde (MDA) were measured by thiobarbituric acid reaction according to the procedure of Ohkawa^21^. Nitric oxide (NO) was measured as nitrite concentration using the method of Ding et al.^22^. Catalase (CAT) activity was estimated according to Aebi^23^. Superoxide dismutase (SOD) activity was determined based on its ability to inhibit the autoxidation of epinephrine in an alkaline medium^24^. Reduced glutathione (GSH) content was estimated using the method of Beutler et al.^25^. All the measured redox parameters were corrected with total protein levels in the testicular homogenate. The pituitary–gonadal hormones were measured using an ELIZA reader (ELx800UV, Bio Tek Instruments, Inc, USA), while the other biochemical parameters were measured using a spectrophotometer (S1200, Unico, USA).

### Histological and histochemical examinations

The testes were promptly fixed in 10% neutral buffered formalin (pH 7.2) for subsequent histological and histochemical examinations. Testis sections were prepared using the paraffin-embedding method. Subsequently, they underwent washing, dehydration in a series of ethanol solutions (ranging from 70 to 100%) to remove water content, and clearing in xylene before embedding in wax. Using a rotary microtome, 5 μm thick sections were cut from the paraffin blocks, followed by deparaffinization in xylene.

The standard staining protocols included ordinary Hematoxylin and Eosin stain for general histological examination^26^, Picro-Sirius red stain for collagen identification^27^, and Periodic acid Schiff (PAS) for demonstrating glycogen content in the testicular tissue^28^. The thickness of the seminiferous tubules' epithelium was measured in micrometers from the basement membrane to the lumen across 10 seminiferous tubules on each slide stained with H&E. Additionally, the number of Leydig cells was counted in 20 random intertubular regions (spaces between three seminiferous tubules) stained with H&E.

### Histopathological scoring

The histopathological assessment examined ten testicular tissue lesions, including vascular congestion, tubular atrophy, necrosis, inflammatory cell infiltration, seminiferous epithelium degeneration, basal membrane thickening, interstitial fibrosis, Leydig cell proliferation, and edema. The findings were categorized into four grades: Grade 0 denoted no observation in the fields, Grade 1 indicated minimal evidence (less than 25% of the fields showed any finding), Grade 2 suggested moderate evidence (25% to 50% of the fields displayed any finding), and Grade 3 represented severe evidence (more than 50% of the fields contained the specified histological parameters). The histopathological scoring was performed according to Sherif et al.^29^.

Examinations and imaging were conducted using a digital camera (Toup Tek ToupView, 2019, Version: × 86, Compatible: Windows XP/Vista/7/8/10, China), ImageJ software, and a computer connected to a light microscope (Olympus CX31, Japan).

### Immunohistochemistry of NF-κB

Following the manufacturer's guidelines, 5 µm thick tissue sections, after deparaffinization, underwent antigen retrieval and were subsequently treated with 3% H_2_O_2_ for 20 min. They were then incubated overnight at 4°C for 30 min with anti-NF-kB p65 antibody (GTX54672, GeneTex Inc., 1:100 dilution). Afterward, sections were washed using immune washing Tris buffer and incubated with the secondary antibody HRP Envision kit (DAKO) for 20 min, followed by another wash using immune washing Tris buffer. The sections were stained with hematoxylin for 2–5 min after establishing the reaction with DAB for 2–3 minutes^30^.

### TUNEL assay

The detection and quantification of apoptosis were performed using an In Situ Cell Death Detection Kit, Fluorescein (Sigma-Aldrich), following the methodology outlined by Waly et al.^31^. This TUNEL technology is based on labeling DNA strand breaks that occur during apoptosis due to the cleavage of genomic DNA.

### Statistical analysis

Data were represented as mean ± SEM. The results were analyzed by one-way analysis of variance (ANOVA) followed by Duncan posthoc test using SPSS program version 16 (SPSS Inc., Chicago, USA). Differences of *p* < 0.05 were considered to be statistically significant.

### Ethics declarations

All experimental approaches were conducted in compliance with the animal care regulations of Assiut University, and were approved by Ethical Committee for Scientific Research at the Faculty of Medicine, Assiut University, Assuit, Egypt (approval number: IRB17300788). The experiment was performed in accordance with the ARRIVE guidelines.

## Results

### Bioactive constituents of ethanolic extract of *Moringa oleifera* leaf using GCMS and HPLC

The GC–MS profile of MLEE revealed the presence of 34 bioactive phytochemical compounds (Table 1). The most abundant compounds identified were 9,19-cyclolanost-24-en-3-ol acetate (12.01%), palmitic acid trimethylsilyl derivative (5.58%), and androstane (3.14%).

**Table 1:** Phytochemical compounds in *Moringa oleifera* leaf ethanolic extract according to GC–MS analysis.

No	Compound	Retention time (minute)	Area%	Matching factor	Molecular formula	Molecular weight
1	à-d-Galactopyranose, 6-O-(trimethylsilyl)-, cyclic 1,2:3,4-bis(methylboronate)	16.37	0.67	655	C11H22B2O6Si	300
2	Phloroglucinol, O,O′-bis(trimethylsilyl)-	20.44	0.59	721	C12H22O3Si2	270
3	5,8,11-Eicosatriynoic acid, tert-butyldimethylsilyl ester	21.54	0.44	697	C26H42O2Si	414
4	à-d-Mannopyranoside, methyl 2,3-bis-O-(trimethylsilyl)-, cyclic butylboronate	21.82	0.26	775	C17H37BO6Si2	404
5	17-Octadecynoic acid	24.27	0.99	719	C18H32O2	280
6	1-Heptatriacotanol	24.75	0.32	751	C37H76O	536
7	1-[2,4,6-tris(trimethylsilox Y)phenyl]-3-[3-methoxy-4-(t rimethylsiloxy)phenyl]-2- propen-1-one	22.17	0.48	871	C28H46O6Si4	590
8	À-d-glucopyranosiduronic acid, 3-(5-ethylhexahydro-2,4,6-t rioxo-5-pyrimidinyl)-1,1-di methylpropyl 2,3,4-tris-o-(trimethylsilyl) -, methyl ester	23.49	0.35	725	C27H52N2O10S i3	648
9	À-d-glucopyranoside, methyl 2-(acetylamino)-2-deoxy-3- O-(trimethylsilyl)-, cyclic methylboronate	24.46	0.46	733	C13H26BNO6Si	331
10	Digitoxin	25.10	0.47	735	C41H64O13	764
11	Methyl glycocholate, 3 trimethylsilyl derivative	25.57	1.01	712	C36H69NO6Si3	695
12	Tristrimethylsilyl ether derivative of 1,25-dihydroxyvitamin D2	25.75	0.63	744	C37H68O3Si3	644
13	Glycine n-[(3à,5á,7à,12à)-24-oxo-3,7,12-t ris[(trimethylsilyl)oxy]ch olan-24-yl]-, methyl ester	26.40	0.73	710	C36H69NO6Si3	695
14	Procyanidin A1	27.61	1.27	944	C30H24O12	576
15	Methyl glycocholate, 3 trimethylsilyl derivative	28.02	0.46	719	C36H69NO6Si3	695
16	Palmitic acid, trimethylsilyl derivative	28.27	5.58	877	C19H40O2Si	328
17	2-(3,4-bis[(trimethylsilyl)oxy]phenyl)-3,5,7-tris[(trimethylsilyl)oxy]-4h-chromen-4-one	29.05	2.29	792	C30H50O7Si5	662
18	Copper phthalocyanine	30.60	2.55	762	C32H16CuN8	575
19	Androstane, 17-(2(5H)-oxofuran-4-yl)-3-(t-butyldi methylsilyloxy)-14-(trimethylsilyloxy)	31.12	3.14	697	C32H56O4Si2	560
20	(2rs)-1,3,8-trimethyl-4-propyl-5-ethyl-2-(1-hydroxyeth Yl)-7-methoxycarbonylethyl-6.gmma.-methylenecarbonyl-porphine	31.78	1.47	585	C36H42N4O4	594
21	Chromozym-tPA	34.66	2.75	937	C24H32N8O7S	576
22	Tetrahydrocannabinolic Acid-A trimethylsilyl derviative	34.78	0.51	697	C28H46O4Si2	502
23	psi.,.psi.-Carotene,3,3',4′,4-tetradehydro-1,1',2,2'-tetrahy dro-1,1'-dimethoxy-2,2'-dioxo-	37.47	0.46	662	C42H56O4	624
24	Androsta–2,4,16-triene-3,6,17-triol, tri-trimethylsilyl	37.57	0.39	709	C28H50O3Si3	518
25	Hecogenin, tert-butyl dimethylsilyl derivative	37.65	0.35	727	C33H56O4Si	544
26	3,9-Epoxypregn-16-en-20-one, 3-methoxy-7,11,18-triacetoxy	38.20	1.79	665	C28H38O9	518
27	á-Sitosterol, trimethylsilyl derivative	38.78	2.62	756	C32H58OSi	486
28	3-Ethenylcholestan-3-ol	38.92	0.56	730	C29H50O	414f.
29	Flavone 4'-oh,5-oh,7-di-o-glucoside	39.76	0.41	773	C27H30O15	594
30	Ethyl iso-allocholate	40.53	1.15	833	C26H44O5	436
31	9,19-Cyclolanost-24-en-3-ol, acetate, (3á)	41.11	12.01	849	C32H52O2	468
32	7,8-Epoxylanostan-11-ol, 3-acetoxy-	42.33	1.78	776	C32H54O4	502
33	9-Octadecenoic acid, (2-phenyl-1,3-dioxolan-4-yl) methyl ester, cis-	42.48	0.29	840	C28H44O4	444
34	Rhodopin	42.71	0.90	771	C40H58O	554

According to HPLC analysis, MLEE contained seven flavonoid components and eight phenolic components. Table 2 presents the detected flavonoid compounds in the extract, with rutin (12.36 μg/gm extract), naringin (11.89 μg/gm extract), and quercetin (10.47 μg/gm extract) identified as the primary compounds. Table 3 displays the phenolic compounds, among which the most abundant were syringenic (12.45 μg/gm extract), cinnamic (11.33 μg/gm extract), and pyrogallol (10.96 μg/gm extract).

**Table 2:** Flavonoids in *Moringa oleifera* leaf ethanolic extract according to HPLC analysis.

Retention time (minute)	Compound	Concentration (μg/gm)
4	Rutin	12.36
5	Naringin	11.89
7	Quercetin	10.47
8	Myricetin	1.23
9	Luteolin	2.65
10	Apigenin	2.33
12	Catechin	3.07

**Table 3:** Phenolic compounds in *Moringa oleifera* leaf ethanolic extract according to HPLC analysis.

Retention time (minute)	Compound	Concentration (μg/gm)
3	Chlorogenic	5.66
5	Syringenic	12.45
6	p-Coumaric	2.16
7	Cinnamic	11.33
9	Pyrogallol	10.96
9.8	Gallic	4.45
11	Ferulic	3.88
13	Benzoic	15.69

### DPPH scavenging activity of ethanolic extract of *Moringa oleifera* leaf

**Figure 1:**
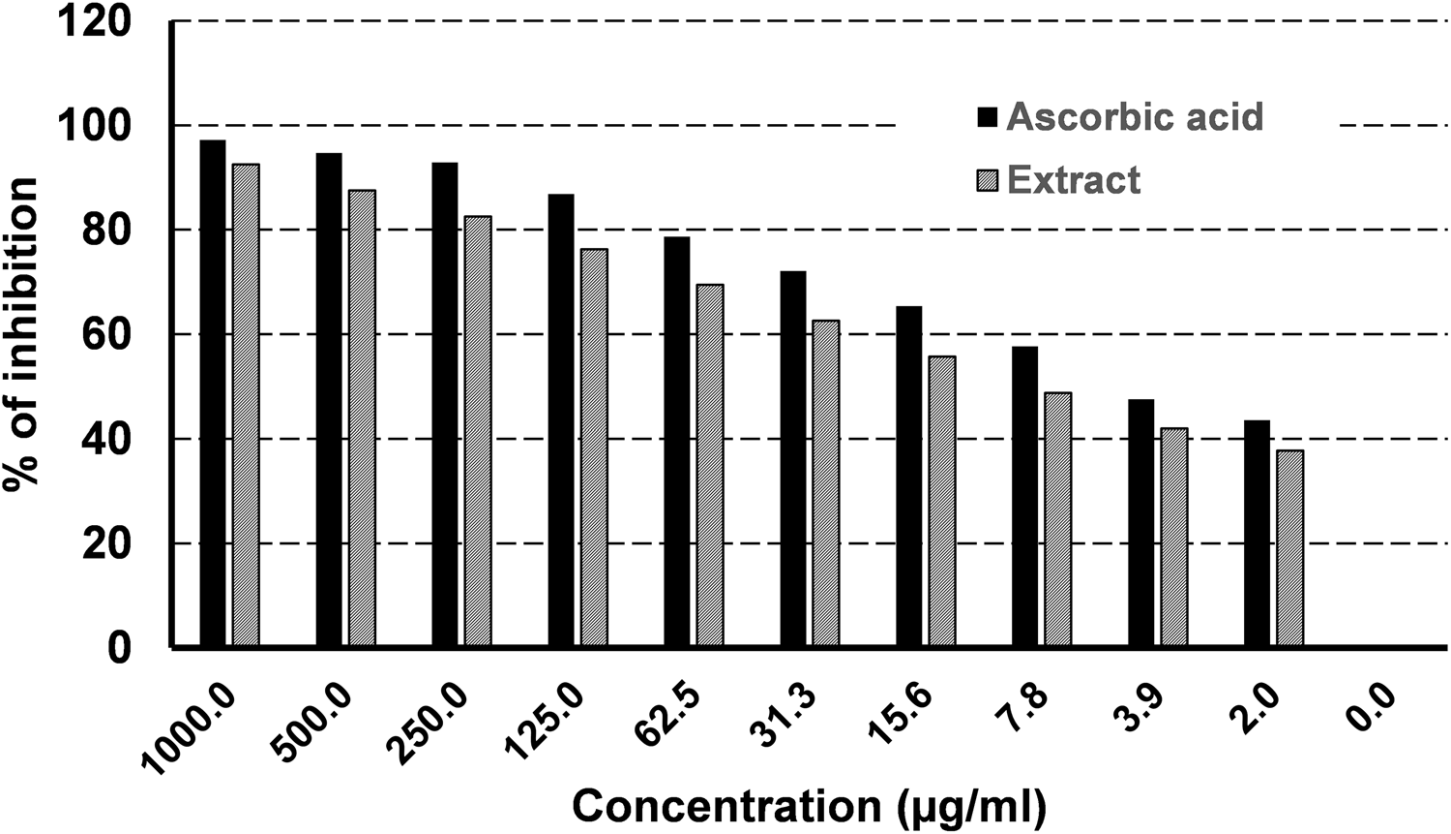
DPPH radical scavenging activity of *Moringa oleifera* leaf ethanolic extract versus ascorbic acid.

The antioxidant activity was determined using the DPPH scavenging activity test with ascorbic acid as a control (Fig. 1). Various concentrations of the extract and ascorbic acid were exposed to the DPPH radical, ranging from 1000 to 1.95 μg/mL. The impact of these concentrations on DPPH radical inhibition was assessed by measuring the absorbance at 517 nm.

Figure 1 illustrates the DPPH radical scavenging ability of the extract and ascorbic acid. The extract demonstrated relatively high antioxidant activity, approaching the antioxidant capacity of ascorbic acid. Specifically, the extract exhibited inhibition percentages of the DPPH radical at 92.5 ± 0.001, 87.5 ± 0.001, and 82.5 ± 0.002 for concentrations of 1000, 500, and 250 µg/mL, respectively, with an IC50 of 2.51 µg/mL. These outcomes closely resemble those obtained with the standard (ascorbic acid), which displayed inhibition percentages of the DPPH radical at 97.14 ± 0.002, 94.86 ± 0.004, and 92.89 ± 0.005 for concentrations of 1000, 500, and 250 µg/mL, respectively, with an IC50 of 1.89 µg/mL.

### *Moringa oleifera* leaf ethanolic extract restored the dynamics of pituitary–gonadal axis and redox equilbrium in uranyl acetate-intoxicated rats

As shown in Table 4, rats exposed to UA exhibited a significant decrease in plasma LH, FSH, testosterone, and E2 levels. However, supplementation with MLEE before UA intoxication reversed these abnormalities, although they did not fully return to the control levels, except for E2, which reverted to its standard state.

**Table 4:** Effects of *Moringa oleifera* leaf ethanolic extract on the plasma pituitary–gonadal hormones and redox parameters of uranyl acetate-intoxicated rats.

Parameter	Group			
	Control	UA	MLEE + UA	*P* value
LH (mU/ml)	11.688 ± 0.905	2.760 ± 0.170^Φ^	6.880 ± 0.644^ΩΨ^	< 0.001
FSH (mU/ml)	24.384 ± 1.918	6.048 ± 0.328^Φ^	14.560 ± 2.201^ΩΨ^	< 0.001
Testosterone (ng/ml)	3.328 ± 0.124	0.816 ± 0.213^Φ^	1.520 ± 0.212^ΩΨ^	< 0.001
E2 (pg/ml)	11.496 ± 0.422	6.816 ± 0.272^Φ^	11.960 ± 0.461^Ω^	< 0.001
MDA (nM/mg protein)	3.150 ± 0.344	6.630 ± 0.393^Φ^	4.650 ± 0.179^ΩΨ^	< 0.001
NO (nM/mg protein)	0.042 ± 0.005	0.024 ± 0.003^Φ^	0.039 ± 0.005^Ω^	0.032
CAT (U/mg protein)	2.010 ± 0.198	0.660 ± 0.077^Φ^	1.260 ± 0.376^ΩΨ^	< 0.001
SOD (U/mg protein)	243.750 ± 27.466	78.660 ± 6.401^Φ^	152.220 ± 7.056^ΩΨ^	< 0.001
GSH (nM/mg protein)	155.195 ± 6.853	77.812 ± 8.380^Φ^	116.844 ± 5.175^ΩΨ^	< 0.001

MLEE: *Moringa oleifera* leaf ethanolic extract; UA: uranyl acetate; LH: luteinizing hormone; FSH: follicle stimulating hormone; E2: 17β-estradiol; MDA: malondialdehyde; NO: nitric oxide; CAT: catalase; SOD: superoxide dismutase; GSH: reduced glutathione.

Results are expressed as the mean ± SEM of six rats per group (One-way ANOVA followed by Duncan post-test).

^Φ^Significant difference between control and UA groups.

^Ω^Significant difference between UA and MLEE + UA groups.

^Ψ^Significant difference between control and MLEE + UA groups.

The redox imbalance induced by UA exposure was apparent through a notable increase in MDA levels and a decrease in NO, CAT, SOD, and GSH levels in the testis of irradiated rats. Oral administration of MLEE to UA-exposed rats effectively reversed these disturbances, although the levels remained below the control levels, except for NO, which returned to normal levels.

### *Moringa oleifera* leaf ethanolic extract improved the histo-architecture of testis in uranyl acetate-challenged rats

**Figure 2:**
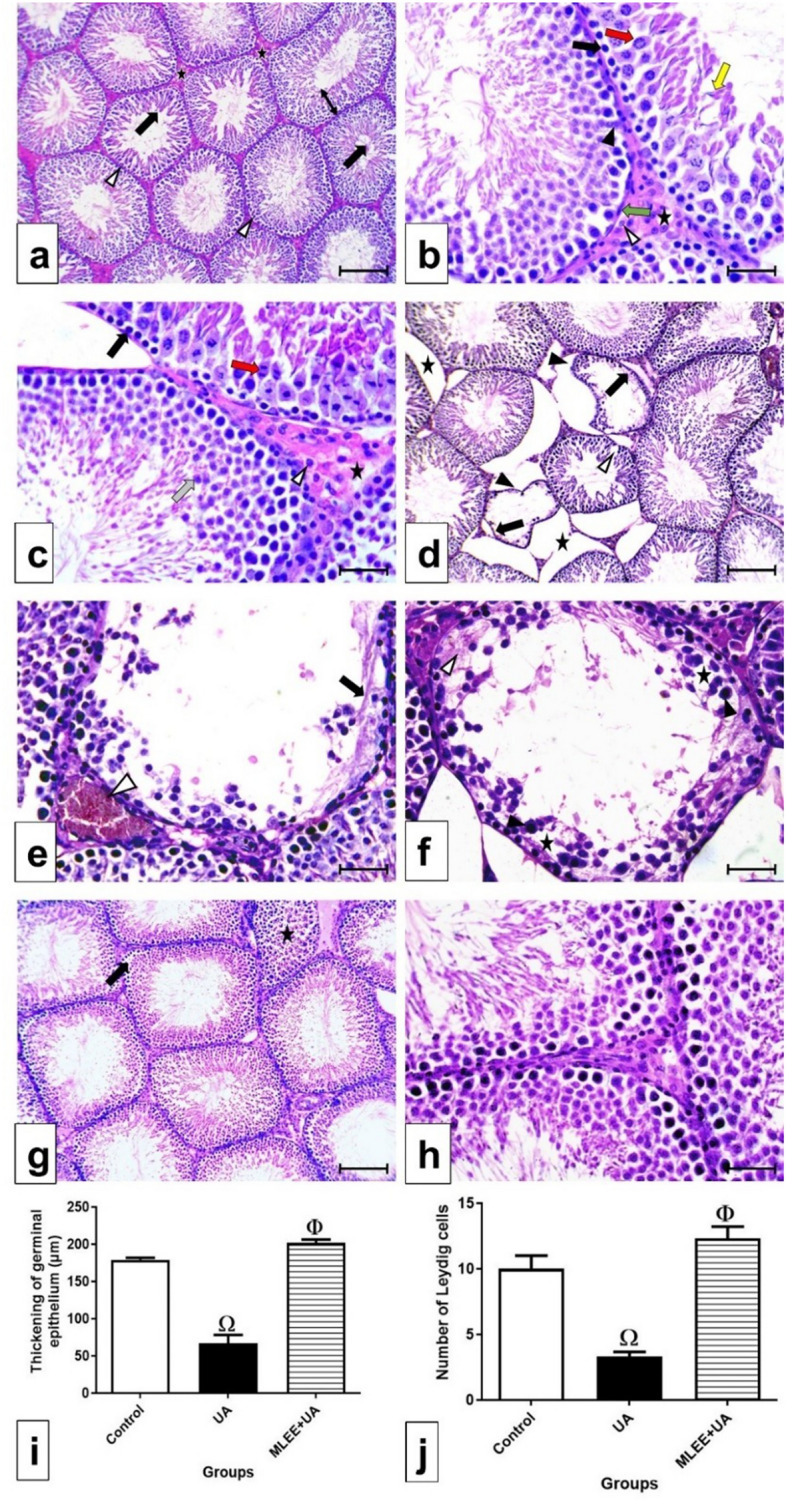
Photomicrographs in testis sections stained by H&E (**a**–**h**), bars = 100 μm (**a**, **d**, **g**) and 50 μm (**b**, **c**, **e**, **f**, **h**). (**a**) In control group showing: closely packed seminiferous tubules with regular basement membranes (Δ), normal thickening of the germinal epithelium (↕) and narrow lumen filled with spermatozoa (black arrow). (**b**, **c**) In control group, interstitial tissue (asterisk) contains Leydig cells (Δ). Myoid cells (green arrow) surround the basement membrane of the tubules. Sertoli cells (black triangle) penetrate the germinal epithelium. Different stages of spermatogenesis appear as: a line of spermatogonia (black arrow) above the basement membrane; primary spermatocytes (red arrow) at different stages of division; spermatids (grey arrow); and spermatozoa (yellow arrow). (**d**) In UA group, showing irregular and atrophied seminiferous tubules (black triangle). Large gaps appear between the tubules (asterisk). Some tubules are with wide lumen free from spermatozoa (black arrow). Spaces between the germ cells are observed (Δ). (**e**) In UA group, showing dilated and congested blood vessel (Δ) and acidophilic remnants of degenerated cells (black arrow) between the few numbers of germ cells (**f**) In UA group, showing disorganized germ cells with condensed nuclei (black triangle). Empty spaces (asterisk) and acidophilic remnants of degenerated cells (Δ) separate the existed germ cells. (**g**) and (**h**) In MLEE + UA group, showing a nearly normal appearance of the seminiferous tubules. They are closely packed with regular cell membranes. The germ cells are organized as normal. Few tubules appear with disorganized germ cells (asterisk) and with spaces in between them (black arrow). (**i**) Thickening of the germinal epithelium (µm) in the different experimental groups. (**j**) Number of Leydig cells in the different experimental groups. Results are expressed as mean ± SEM (One-way ANOVA followed by Duncan post-test). Ω significant difference between UA and the control groups. Φ significant difference between MLEE + UA and UA groups.

In the control group (Fig. 2a–c), hematoxylin and eosin-stained sections of the testis displayed a normal structure. The closely packed rounded seminiferous tubules were surrounded by a regular basement membrane and enveloped in flat-nucleated myoid cells. Blood vessels and Leydig cells were typically observed in the interstitial tissue between the tubules. Within the seminiferous tubules, two types of cells were evident: the germinal epithelium and Sertoli (support cells). The germinal epithelium comprised the spermatogenic lineage, beginning with a row of spermatogonia resting on the basement membrane. Sections often depicted primary spermatocytes at various division stages, while secondary spermatocytes, due to their rapid division, were scarcely found. Spermatids, resembling small, round cells with round, vesicle-shaped nuclei near the center, and mature sperm, characterized by long nuclei connected to Sertoli cells by their heads, filled the seminiferous tubules' lumen.

In the UA treated group (Fig. 2d–f), the seminiferous tubules appeared irregular, atrophied, and loosely packed, resulting in widened spaces within the interstitial tissue containing dilated and congested blood vessels. Some tubules lacked germ cells, leading to widened lumens devoid of spermatozoa. The remaining germ cells exhibited disorganization with dense nuclei, and wide spaces separated them. Acidophilic remnants of degenerated cells were evident. Morphometric analysis revealed significant thickening of the germinal epithelium and a decreased number of Leydig cells (*P* < 0.001) in the UA group compared to the control group, as shown in Fig. 2i, j.

In the MLEE + UA group (Fig. 2g, h), the seminiferous tubules appeared nearly normal, being closely packed with a regular basement membrane. Their lumens were filled with spermatozoa, and the germ cells exhibited normal arrangement. Morphometrically, a significant increase in the thickening of the germinal epithelium and the number of Leydig cells (*P* < 0.001 and *P* < 0.01, respectively) was observed in the MLEE + UA group compared to the UA group. However, insignificant differences were observed versus those of the control group, as depicted in Fig. 2i, j. The scores of histopathological lesions in the testes of the examined groups were illustrated in Table 5.

**Table 5:** The score of histopathological lesions in the examined groups.

Lesions	Groups		
	Control	UA	MLEE + UA
Vascular congestion	0	2	0
Vascular dilatation	0	3	0
Tubular atrophy	0	2	1
Necrosis	1	3	1
Inflammatory cells infiltration	1	1	0
Degeneration of seminiferous epithelium	0	2	0
Basal membrane thickening	0	0	1
Interstitial fibrosis	0	2	1
Leydig cells proliferation	0	0	1
Edema	0	1	0

*MLEE*:*Moringa oleifera* leaf ethanolic extract; UA: uranyl acetate.

(0) Absent lesion. (1) Slight (< 25% of the field contained the lesion). (2) Moderate (from 25 to 50% of the field contained the lesion). (3) Severe (> 50% of the field contained the lesion).

### *Moringa oleifera* leaf ethanolic extract alleviated the collagen deposition in the testis of uranyl acetate-challenged rats

**Figure 3:**
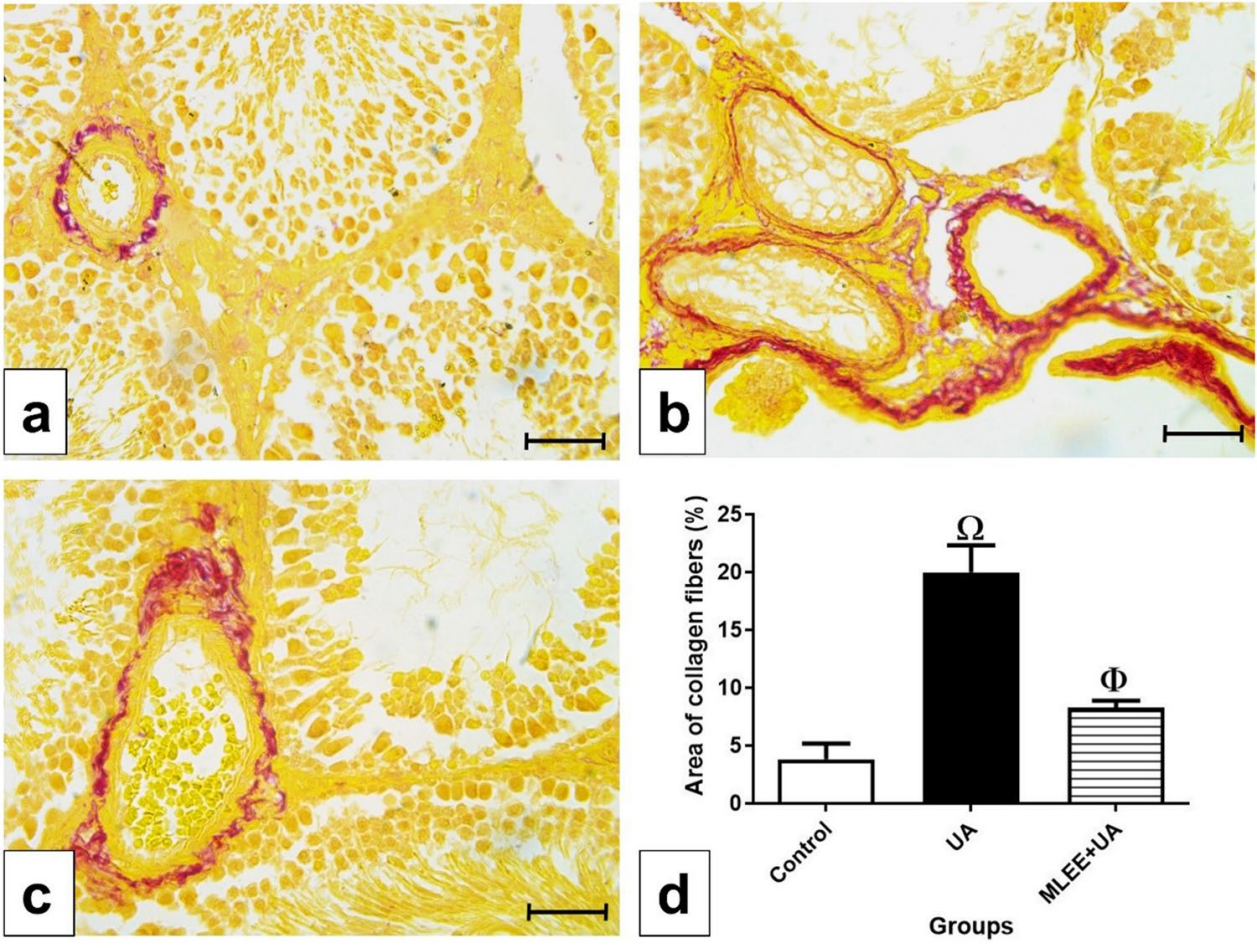
Examination of collagen fibers in the experimental groups. (**a**–**c**) photomicrographs of testis sections stained by Picro-Sirius red stain, bar = 50μm. (**a**) In control group, showing tiny amount of collagen fibers around the blood vessel. (**b**) In UA group, showing high amount of collagen fibers around the blood vessels and in between them represented by the red color. (**c**) In MLEE + UA group, showing moderate amounts of collagen fibers. (**d**) Percentage of area of collagen fibers in the different experimental groups. Results are expressed as mean ± SEM (One-way ANOVA followed by Duncan post-test). Ω significant difference between UA and the control groups. Φ significant difference between MLEE + UA and UA groups.

In the control group, examination of collagen fibers using Picro-Sirius red stain revealed minimal amounts of collagen fibers around the blood vessels (Fig. 3a). Conversely, in the UA group, an abundance of collagen fibers was evident, indicated by the prominent red color around and between the blood vessels (Fig. 3b). Statistically, the increase in the percentage of collagen area in the UA group was significant (*P* < 0.001) compared to the control group (Fig. 3d). In the MLEE + UA group, the presence of collagen fibers around the blood vessels was reduced compared to the UA group (Fig. 3c), and this decrease was statistically significant (*P* < 0.01). The representation of collagen area percentages in the different experimental groups is shown in Fig. 3d.

### *Moringa oleifera* leaf ethanolic extract normalized the glycogen content in the testis of uranyl acetate-challenged rats

**Figure 4:**
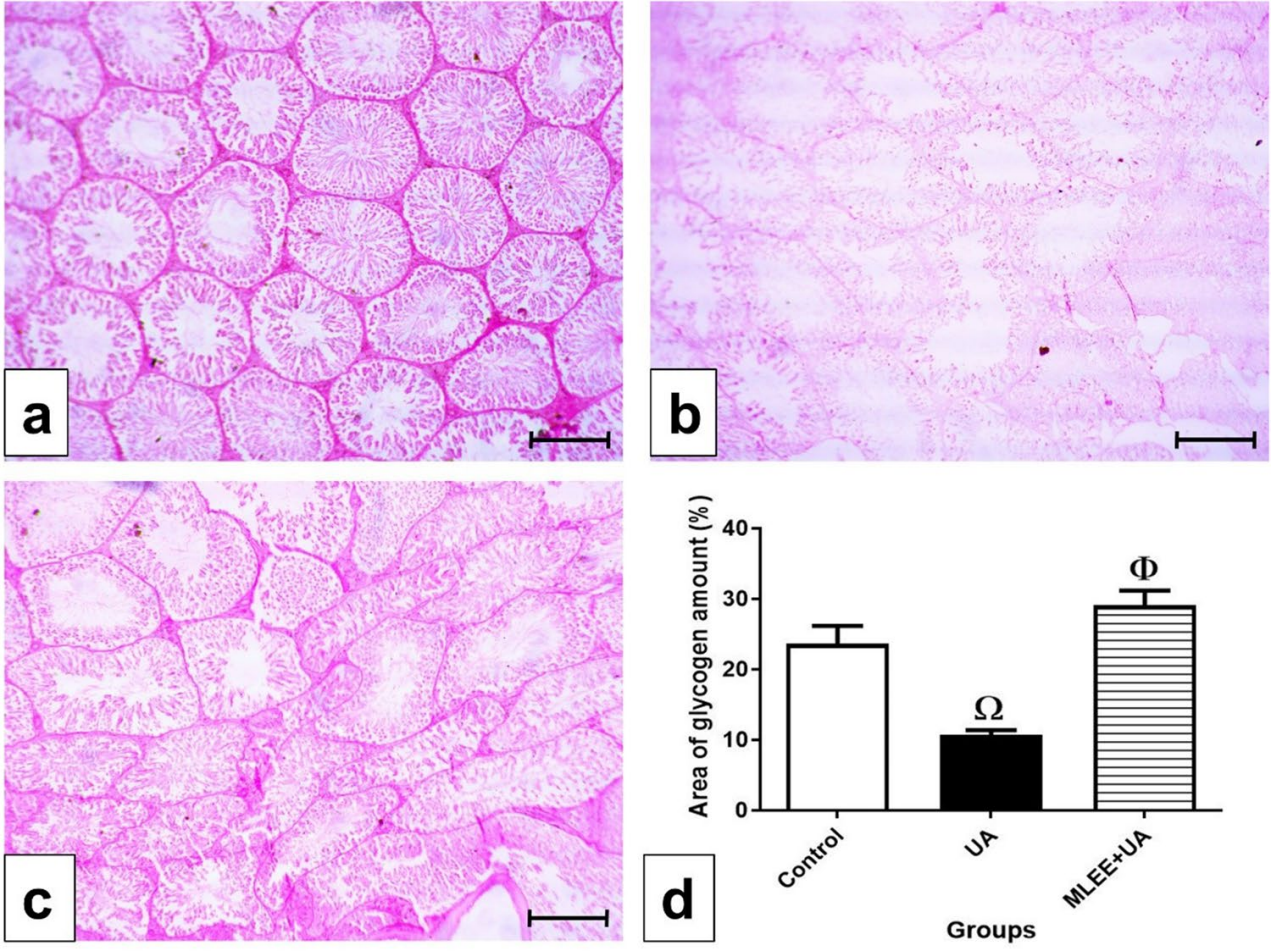
Examination of glycogen amount in the experimental groups. (**a**–**c**) photomicrographs of testis sections stained by Periodic acid–Schiff stain (PAS), bar = 100 μm. (**a**) In control group, high glycogen content as a positive PAS reaction in the basement membranes and the interstitial tissue. (**b**) In UA group, showing marked depletion of the glycogen content in the interstitial tissue and the basement membranes of the seminiferous tubules. (**c**) In MLEE + UA group, showing positive PAS reaction resembling those of control group. (**d**): Percentage of area of glycogen amount in the different experimental groups. Results are expressed as mean ± SEM (One-way ANOVA followed by Duncan post-test). Ω significant difference between UA and the control groups. Φ significant difference between MLEE + UA and UA groups.

In the control group, examination of glycogen content using Periodic acid-Schiff stain (PAS) revealed high glycogen levels, indicated by a positive PAS reaction observed in the basement membranes and interstitial tissue (Fig. 4a). However, in the UA group, a marked depletion of glycogen content was evident in both the interstitial tissue and the basement membranes of the seminiferous tubules (Fig. 4b). This decrease was statistically significant (*P* < 0.01) compared to the control group.

In the MLEE + UA group, a positive PAS reaction resembling that of the control group was observed (Fig. 4c). The increase in glycogen content was statistically significant (*P* < 0.01) when compared to the UA group but showed no significant difference compared to the control group. The representation of the percentage of glycogen area in the different experimental groups is depicted in Fig. 4d.

### *Moringa oleifera* leaf ethanolic extract normalized the immuno-expression of NF-kB in the testis of uranyl acetate-challenged rats

**Figure 5:**
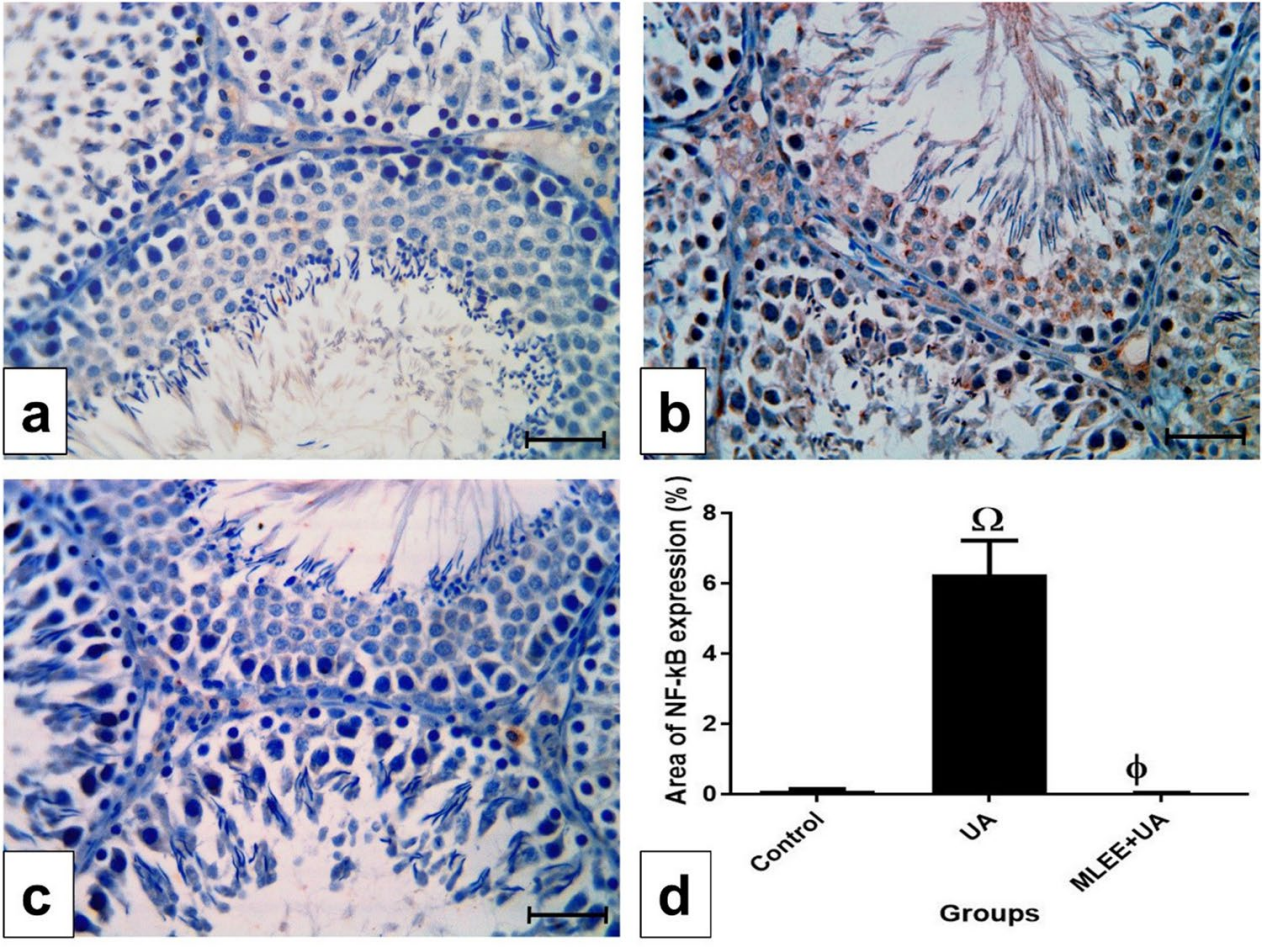
Immunohistochemical detection of NF-kB in the testis. (**a**–**c**) Photomicrographs of testis sections of rats from the experimental groups, bar = 50μm. (**a**) In control group, showing a negative immunoreaction of NF-kB. (**b**) In UA group, showing positive immunoreaction of NF-kB as represented by the brown color (**c**) In MLEE + UA group, showing negative immunoreaction. (**d**) Percentage of area of NF-kB expression in the different experimental groups. Results are expressed as mean ± SEM (One-way ANOVA followed by Duncan post-test). Ω significant difference between UA and the control groups. Φ significant difference between MLEE + UA and UA groups.

In the control group, a negative immunoreaction of NF-kB was observed (Fig. 5a). In contrast, the UA group exhibited a positive immunoreaction of NF-kB, indicated by the brown coloration (Fig. 5b). However, in the MLEE + UA group, a negative immunoreaction was observed (Fig. 5c). The representation of the percentage area of NF-kB protein expression in Fig. 5d demonstrated that UA administration significantly increased the expression of NF-kB compared to the control group (*P* < 0.001). Conversely, Moringa intervention reduced NF-kB expression, resembling the levels observed in the control group.

### *Moringa oleifera* leaf ethanolic extract exerted an anti-apoptotic effect against uranyl acetate-induced testicular dysfunction rats

**Figure 6:**
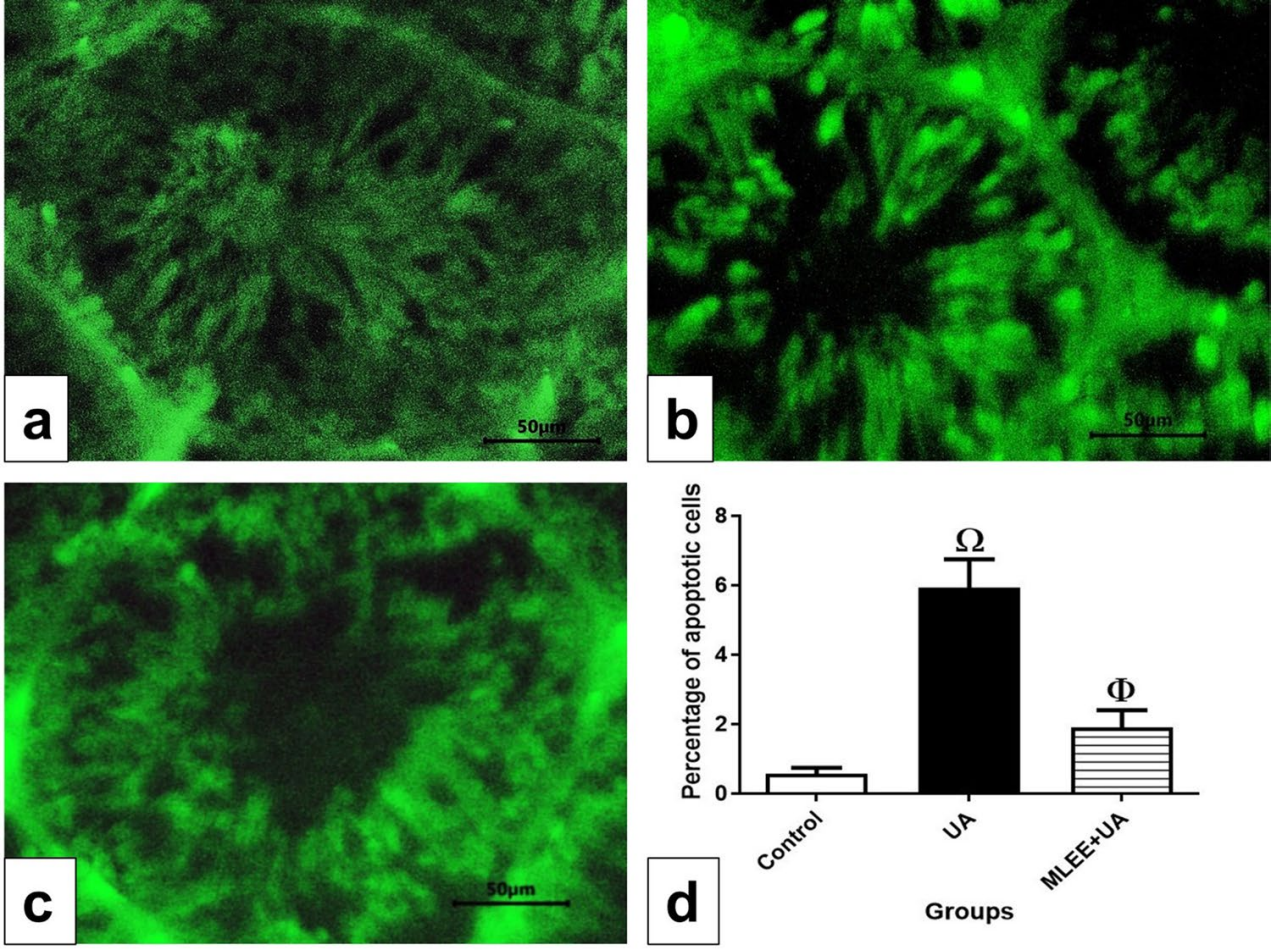
Fluorescent photomicrograph of TUNEL assay in paraffin sections showed the protective effect of MLEE on UA-induced testicular damage in rats. Results are expressed as the mean ± SEM of six rats per group (One-way ANOVA followed by Duncan post-test). ^Φ^Significant difference between control and UA groups. ^Ω^Significant difference between UA and MLEE + UA groups.

We employed a TUNEL assay on paraffin sections to detect apoptosis. In the control group, a few apoptotic spermatogenic cells were observed (Fig. 6a), whereas the UA group exhibited a high number of apoptotic spermatogenic cells (Fig. 6b). Conversely, the MLEE + UA group displayed a reduced number of apoptotic spermatogenic cells (Fig. 6c), similar to the control. The exposure to UA significantly increased the percentage of apoptotic cells compared to the control group (*P* < 0.001). However, the administration of MLEE prior to UA toxicity reduced the apoptotic cell count, restoring it to the control level (Fig. 6d).

## Discussion

The challenge posed by UA has been linked to negative modulation in the pituitary–gonadal axis, as indicated in prior reports^32,33^. This outcome is associated with the down-regulation of genes encoding enzymes involved in steroid production, signal transduction, and LH receptor activity^34,35^. Acting as a xenoestrogenic compound, UA disrupts estrogen receptors, affecting their production and metabolism^36^. Uranyl nitrate alters the secretion of hypothalamic biogenic amines like dopamine, accumulating in the adult rat striatum^37,38^. Considering that the hypothalamus and striatum are in close proximity within the brain, it's hypothesized that UA disrupts endocrine function by negatively impacting GnRH secretion and receptor activity.

Peroxidative injury to cellular macromolecules plays a significant role in the etiology and progression of testicular dysfunction. The substantial accumulation of free radicals disrupts the hormonal balance governing male genital activity by influencing the hypothalamic-pituitary-gonadotropic axis, consequently reducing anterior pituitary release of LH and FSH^39^. Restricted LH production leads to Leydig cell failure in generating sufficient testosterone^40^. Simultaneously, reduced FSH impacts androgen-binding protein discharge, affecting testosterone concentration stability. This impact on Leydig cell synthetic ability influences proteins controlling cholesterol influx into the mitochondria, such as steroidogenic acute regulatory (StAR) protein^41^.

The protective effects of MLEE against the reduction in reproductive hormones before UA exposure align with observations in a tramadol-intoxicated rat model^9^. Additionally, an aqueous extract of MO leaves significantly increased serum testosterone levels and gene expressions of LH and FSH in rabbit bucks^8^. MLEE has been shown to up-regulate genes such as CYP11A1, hydroxysteroid 17-beta dehydrogenase 3, StAR, and CYP17A^42,43^. The abundance of biologically active ingredients in MLEE, as evident from HPLC and GCMS analyses, contributes to rebalancing the sexual endocrine system, improving testicular redox status, and mitigating apoptotic manifestations and histopathological lesions^44^.

Flavonoids like quercetin, apigenin, and luteolin have been reported to boost Leydig cell activity, enhancing testosterone production by upregulating steroidogenic proteins^45,46^. Furthermore, catechin has been found to potentiate the stimulatory effects of gonadotropin-releasing hormone on LH release and human chorionic gonadotropin on testosterone release by Leydig cells in vitro^47^.

The significant increase in testicular MDA and decrease in enzymatic and non-enzymatic antioxidants in the UA group mirror findings in renal tissue^20^, but differ from those reported in testicular tissue^48^. These contrasting responses are likely due to variations in UA doses; for instance, the earlier research group used a dose of 1 mg/kg body weight compared to our experimental protocol of 5 mg/kg body weight. UA's interference with the respiratory chain leads to excessive reactive oxidant production, accelerating the lipid peroxidation cascade^49^. Alternatively, oxidative stress triggers the activation of Notch and transforming growth factor β pathways, leading to the excessive accumulation of fibrotic proteins and collagen^50^. Moreover, it may induce fibroblast transdifferentiation to myofibroblasts, a crucial step in fibrosis^51^.

The decline in FSH output in the UA group could contribute to inducing testicular redox disturbance, given gonadotropic hormones' ability to counteract oxidative damage^52,53^. There exists a reciprocal relationship between redox imbalance and testosterone deficiency. The overproduction of free radicals inhibits transcription factors involved in the expression of several steroidogenic enzymes^54^. Oxidative stress suppresses phosphorylation of StAR protein, a key enzyme in testosterone production, limiting its translocation into mitochondria^55^. This stress also induces mitochondrial membrane depolarization, linked to post-transcriptional inhibition of the steroidogenic acute regulatory protein^56^. Conversely, testosterone depletion activates reactive oxygen species production by interfering with mitochondrial function^57,58^. The notable increase in testicular NO underscores the positive regulation of iNOS expression at both transcriptional and post-transcriptional levels by NF-κB^59^.

We, along with other researchers^12,60^, have confirmed the free radical scavenging properties of MLEE and the presence of several antioxidant phenolic and flavonoid phytochemicals. These chemobiological attributes mitigate the need for an amplified antioxidant network shield and redox-sensitive transcription factors, aiding in the restoration of the oxidant/antioxidant equilibrium. The increase in SOD, CAT, and GSH levels in the testis of UA-intoxicated rats following MLEE supplementation aligns with observations in rats affected by aluminum^61^ and acrylamide^62^.

The histopathological perturbations observed in our investigation align with earlier studies suggesting that UA exposure triggers adverse cytological alterations in the testis^48,63^. Inhibition of enzymes associated with sperm synthesis, maturation, and energy metabolism^32,33^ might contribute to the presence of azoospermia in some seminiferous tubules. The reduction in FSH leads to spermatogenic disorders, given its vital role in initiating and maintaining spermatogenesis and nurturing developing germ cells by Sertoli cells^64,65^. The compromised functional integrity of the reproductive axis leads to the thickening of the germinal epithelium and atrophy of seminiferous tubules, as its hormones are essential for preserving the structural morphology of the testicular microenvironment^66^. Flavonoids present in MLEE, such as luteolin, preserve seminiferous tubules and blood-testis barrier stability by enhancing the expression of numerous downstream antioxidant genes and increasing the protein expression of ZO-1, occludin, claudin-11, and Cx43^67^. These compounds possess the ability to permeate the lipid bilayer of membranes, providing direct protection for spermatozoa against peroxidative damage, while also stimulating the electron transport chain and oxidative phosphorylation to energize the germ cells^68,69^. Histomorphometric measurements revealed a resurgence in germinal activity in the MLEE + UA group, consistent with findings reported by Laoung-On et al.^70^.

The depletion of testicular glycogen reserves observed in the UA group aligns with findings in the liver of UA-intoxicated rats^71^, suggesting an attempt to allocate more energy for combating oxidative stress^72^. Conversely, the augmented glycogen stores observed in the testis of the MLEE + UA group mirror previous observations in the liver and muscle of rats subjected to a forced swimming endurance test^73^, potentially attributable to increased glycogen synthase activity, glycogen storage, and glucose uptake^74^. Compounds like gallic acid, p-coumaric acid^75^, and quercetin^76^ likely facilitate cellular glucose incorporation and glycogenesis, potentially due to the enhancement of beta-cell efficiency.

Consistent with the observed collagen accumulation in the testicular tissue of the UA group, Zhu et al.^77^ noted interstitial fibrosis in the renal tissues following long-term implantation of gastrocnemius muscle fragments in rats with UA. MLEE succeeded in alleviating collagen deposition in the testes of UA-challenged rats, akin to its effects in acetaminophen-induced liver fibrosis^78^. This effect may be attributed to the decrease in gene expression of tumor necrosis factor, which plays an important role in activating the major fibrogenic molecule transforming growth factor-β and stimulating the survival and production of activated myofibroblasts^79^. Compounds like flavonoids and saponins have been shown to down-regulate fibrosis-related gene expression^80–82^.

The significant increase in apoptotic immuno-stained cells observed in the testes of the UA group aligns with previous findings^48^, indicating disturbances in energy homeostasis and cytolysis in the mitochondrial outer membrane, ultimately leading to the release of apoptotic mediators^49,83^. Reactive oxygen species up-regulate genes encoding redox-regulated transcription factors like c-Jun N-terminal kinase, known to be associated with the initiation of apoptosis^84^. A reduced Bcl-2/Bax ratio under UA burden shifts cell programming towards decisions of cell death^84^. The elevation in testicular NO levels closely relates to cell death, as NO enhances Fas-mediated apoptosis^85^. GSH depletion leads to intracellular glutathione efflux, associated with increased caspase-3 activity due to a reduction in extracellular GSH^86^. The reduced secretion of LH creates conditions conducive to cell destruction, as LH is considered an agent that blocks apoptosis^87^.

MLEE intervention successfully mitigated cell death processes in the testes, similar to observations in cyclophosphamide-intoxicated mice^88^. This intervention down-regulated the gene expression of caspase-3 and Bax while up-regulating Bcl-2 gene expression, enhancing mitochondrial membrane potential^89,90^, leading to MLEE's anti-apoptotic effects. Phenolic compounds like p-coumaric acid mitigated alcohol-induced male reproductive impairments in rats by reducing the immunoreactivity of caspase-3, caspase-7, and p21^91^. Ferulic acid antagonizes calcium influx, reactive oxygen emission, and cytochrome c-mediated caspase-3-dependent apoptosis^92,93^. MLEE exhibits radioprotective properties by enhancing the transcript levels of proliferating cell nuclear antigen, favoring cellular survival over suicide signals^94^. The increased repair of DNA damage is suggested by rutin's activation of ataxia telangiectasia mutated, a critical factor in resolving double-stranded DNA breaks^95^.

Consistent with our findings and in line with previous research^20^, UA exposure elevated the testicular immuno-expression of NF-кB. Factors contributing to peroxidative stress trigger the phosphorylation of inhibitor kappa B, leading to its proteasomal degradation, allowing the NF-kB free P65 subunit to translocate into the nucleus^96^. The down-regulation observed in the immuno-expression of testicular NFкB in the MLEE + UA group is similar to observations in the liver of lead acetate-challenged rats^97^. Additionally, research has shown that naringin inhibits the NF-κB signaling pathway in sepsis-induced intestinal injury in mice^98^.

The study was conducted for a limited period (14 days), which might not capture the long-term effects of MLEE supplementation or UA exposure. The study used a specific number of rats; however, larger sample sizes could enhance statistical robustness and reliability of the findings. The study administered a single dose of UA, which might not replicate the complexity of chronic exposure scenarios or different dosage effects. While the study provides valuable insights into the potential protective effects of MLEE, a deeper mechanistic understanding of how MLEE specifically interacts with UA-induced toxicity pathways is warranted. The research focused on specific parameters related to oxidative stress, hormonal changes, apoptosis, and histopathology. Incorporating additional biomarkers or comprehensive assessments might offer a more holistic view of the impact and mechanisms involved. While animal models provide essential insights, translating these findings directly to human applications requires further clinical investigations. Addressing these limitations in future studies could further validate and broaden the understanding of potential protective role of MO against UA-induced testicular toxicity.

## Conclusion

Our findings confirm the testis' susceptibility to UA intoxication on both biochemical and cytological levels. Hence, it's crucial to pay specific attention to the potential impacts of UA on the reproductive aspects of human populations residing near contaminated areas. MLEE offers a relatively safe and cost-effective approach to counteracting gonadotoxic radiological agents. Further studies are highly recommended to explore the potential protective effects of MLEE against other health hazards induced by UA.

## Data Availability

The datasets analyzed during the current study available from the corresponding author on reasonable request.
